# Increased Serum Parathyroid Hormone, Osteocalcin and Alkaline Phosphatase Are Associated with a Long-Term Adverse Cardiovascular Outcome after Coronary Artery Bypass Graft Surgery

**DOI:** 10.3390/diagnostics9040143

**Published:** 2019-10-08

**Authors:** Olga Barbarash, Mikhail Zykov, Vasiliy Kashtalap, Oksana Hryachkova, Alexandr Kokov, Olga Gruzdeva, Irina Shibanova, Anton Kutikhin

**Affiliations:** 1 Research Institute for Complex Issues of Cardiovascular Diseases, Kemerovo 650002, Russia; barbol@kemcardio.ru (O.B.); mvz83@mail.ru (M.Z.); kashvv@kemcardio.ru (V.K.); hrychon@kemcardio.ru (O.H.); kokoan@kemcardio.ru (A.K.); gruzov@kemcardio.ru (O.G.); shibia@kemcardio.ru (I.S.); 2 Department of Cardiology and Cardiovascular Surgery, Kemerovo State Medical University, Kemerovo 650056, Russia

**Keywords:** osteopoenia, osteoporosis, coronary atherosclerosis, coronary artery disease, adverse outcome, bone turnover markers, ionised calcium, osteocalcin, parathyroid hormone, alkaline phosphatase

## Abstract

Despite the fact that an association of osteopoenia/osteoporosis with elevated risk of coronary artery calcification (CAC) and coronary atherosclerosis (CA) is well-established, it remains unclear whether bone turnover markers can be employed in long-term prognostication of such patients. Here we measured serum calcium, phosphate, calcitonin, parathyroid hormone (PTH), osteoprotegerin, osteocalcin, osteopontin, alkaline phosphatase and its bone isoenzyme, subsequently correlating them with an adverse cardiovascular outcome after 3 years of follow-up. The extent of brachiocephalic artery stenosis, CA, or CAC, as well as prevalence of osteopoenia/osteoporosis before the coronary artery bypass graft (CABG) surgery, did not differ between outcome groups, suggesting that subtle molecular mechanisms might be involved in determining the outcome rather than clinical or subclinical disease. After stepwise logistic regression, serum osteocalcin > 26.8 ng/mL and PTH > 49.1 pg/mL were independent predictors of an adverse outcome. Serum ionised calcium correlated with multivessel coronary artery disease; moreover, patients with severe CA (SYNTAX score > 21) had higher serum ionised calcium than those with mild CA. Likewise, serum alkaline phosphatase was associated with severe CA and CAC (Agatston score > 400). In conclusion, serum PTH, osteocalcin, and alkaline phosphatase are associated with an adverse cardiovascular outcome 3 years after CABG surgery regardless of osteopoenia/osteoporosis, coronary/peripheral atherosclerosis, and CAC.

## 1. Introduction

Current advances in the treatment of coronary artery disease (CAD) are mainly related to myocardial revascularisation. Coronary artery bypass graft (CABG) surgery significantly improves prognosis and quality of life in all patients with CAD, particularly in those with high cardiovascular risk [1]. However, angina pectoris is a prevalent (around 25%) long-term complication of CABG surgery due to a graft occlusion or coronary atherosclerosis (CA), both of which do frequently occur in comorbid patients; hence, screening for the markers of an adverse cardiovascular outcome is rapidly ongoing [2,3].

Coronary artery calcification (CAC) is a major risk factor of cardiovascular events and death in general population [4], as well as patients with chronic kidney disease (CKD) [5,6] or CAD [7]. Further, breast arterial calcification is also independently associated with an increased risk of all-cause and cardiovascular death [8]. In addition, CAC correlates with a lower efficacy of CABG surgery [9]. Extraskeletal calcification is to a large extent similar to bone tissue formation [10], yet its mechanisms are still under scrutiny. Despite osteopoenia/osteoporosis is associated with both CAC and CA [11], prognostic significance of bone turnover markers in patients with stable angina pectoris (SAP) after CABG surgery has been scarcely evaluated.

## 2. Materials and Methods 

The study sample consisted of 111 consecutive male patients admitted to Research Institute for Complex Issues of Cardiovascular Diseases in 2015. Criteria of inclusion were CABG surgery due to CCS grade I–III SAP, age < 75 years, and a written informed consent to participate in the study. Criteria of exclusion were progression of SAP to CCS grade IV, New York Heart Association class IV chronic heart failure, past medical history of CABG surgery, diagnosed acute/chronic liver or kidney failure, cancer, chronic obstructive pulmonary disease, autoimmune, endocrine (excluding type 2 diabetes mellitus), mental, blood, and/or severe digestive disorders, alcoholism, obesity (defined as body mass index ≥ 30 kg/m^2^), and use of glucocorticoids > 3 months all documented from the medical records. The investigation was carried out in accordance with the Good Clinical Practice and the Declaration of Helsinki. The study protocol was approved by the Local Ethical Committee of Research Institute for Complex Issues of Cardiovascular Diseases (ethical approval code 44025, approved on 12 December 2014). All patients provided written informed consent after receiving a full explanation of the study. 

SAP, chronic heart failure, arterial hypertension, and type 2 diabetes mellitus were diagnosed according to the respective European Society of Cardiology guidelines [12,13,14,15] while overweight/obesity was defined as recommended in National Institute for Health and Care Excellence guidelines [16]. Glomerular filtration rate was calculated according to Modification of Diet in Renal Disease formula. Past medical history of MI, stroke, and smoking status were defined using the medical records. 

Echocardiography (Sonos 2500, Hewlett Packard), color duplex screening of brachiocephalic arteries (Vivid 7 Dimension, General Electric Healthcare), coronary angiography (Innova 3100, General Electric Healthcare), multislice spiral computed tomography (SOMATOM Sensation 64 and Leonardo multimodality workstation, Siemens Healthcare), and dual-energy X-ray absorptiometry (Norland XR-46, Orthometrix) were performed in all patients before the CABG surgery. The results of coronary angiography and multislice spiral computed tomography were evaluated using SYNTAX score to measure the severity of CA and Agatston score to quantitate CAC, respectively. The results of dual-energy X-ray absorptiometry were interpreted according to the International Society for Clinical Densitometry Official Positions [17]. Bone mineral density of ≤ 1 standard deviation below the mean peak bone mass (average of healthy young adult male) at the lumbar spine (L_I_–L_IV_) and femoral neck was considered as normal. Osteopoenia and osteoporosis were defined as a bone mineral density of 1–2.49 and ≥ 2.5 standard deviations below the mean peak bone mass, respectively. Clinicopathological features of the patients are indicated in Table 1.

During the hospital stay, all patients received the standard therapy of antiplatelet drugs, beta-blockers, angiotensin-converting enzyme inhibitors or angiotensin receptor II blockers, and lipid-lowering drugs which were also prescribed to them before hospital discharge. Follow-up was performed by an outpatient visit in 91 (82%) patients; other individuals were unavailable due to the change of phone number or place of residence. Study endpoints included progression of AP, MI, or cardiovascular death. 

Measurement of serum calcium, phosphate, and alkaline phosphatase was performed before the CABG surgery using the respective kits (Biosys Health) and KONELAB 320i biochemical analyzer (Thermo Scientific) (Table 2). Serum bone alkaline phosphatase, calcitonin, PTH, osteocalcin, osteoprotegerin, and osteopontin was quantified by an enzyme-linked immunosorbent assay using the respective kits of Metra Biosystems (8012), Biomerica (7024 and 7022), IDS (AC-11F1), Invitrogen (BMS2021INST), and Enzo Life Sciences (ADI-900-142) (Table 2).

Statistical analysis was carried out utilising SPSS 22 (IBM). A sampling distribution was assessed by Shapiro–Wilk test. Descriptive data were represented by proportions, median and interquartile range (25th and 75th percentiles). Intergroup differences were evaluated by Mann–Whitney U-test. To assess the correlation, Spearman’s rank correlation coefficient, as well as enter and stepwise logistic regression, were employed. Threshold values for logistic regression equations were selected according to the highest quartile in patients having favorable outcome. *p* values ≤ 0.05 were regarded as statistically significant.

## 3. Results

We consecutively recruited 111 male patients who underwent CABG surgery due to Canadian Cardiovascular Society (CCS) grade I–III SAP; follow-up was carried out by an outpatient visit and was successful in 91/111 (82%) patients. After 3 years of follow-up, 70 out of 91 (76.9%) patients did not have any symptoms of CAD, while 16 (17.6%) suffered from recurrent CCS class I–II SAP. Cardiovascular death was registered in 4 (4.4%) patients due to myocardial infarction (MI) (3/4) or stroke (1/4) while 1 (1.1%) patient experienced non-fatal MI. There were no differences in the extent of brachiocephalic artery stenosis, CA, or CAC as well as in prevalence of osteopoenia/osteoporosis before the CABG surgery in patients with favorable and adverse cardiovascular outcome (Table 3). Post-discharge treatment was also similar and included antiplatelet drugs, beta-blockers, angiotensin-converting enzyme inhibitors/angiotensin receptor II blockers, and lipid-lowering drugs.

Regarding the parameters of mineral homeostasis, adverse cardiovascular outcome was not associated with preoperative serum calcium, phosphate, calcitonin, or parathyroid hormone (PTH) (Table 4). Yet, patients with an adverse outcome had 2.2-, 1.5-, and 1.2-fold higher serum alkaline phosphatase, osteopontin, and osteocalcin along with 1.6-fold lower osteoprotegerin as compared to those with a favorable outcome (Table 4).

To examine the differences in the parameters of mineral homeostasis in patient subgroups, we further stratified the study participants in relation to body mass index and type 2 diabetes mellitus. Similar to the patients with adverse outcome, those with overweight had 1.35-fold higher serum osteocalcin as compared to the subjects with normal body mass index (Table 5), while no statistically significant differences have been found in patients with or without type 2 diabetes mellitus (Table 6).

Multivariate analysis confirmed that brachiocephalic artery stenosis, CA, CAC, and osteopoenia/osteoporosis were not significant predictors of an adverse outcome in contrast to preoperative levels of serum osteopontin > 8.3 ng/mL, PTH > 49.1 pg/mL, smoking, alkaline phosphatase > 186 U/L, and osteocalcin > 26.8 ng/mL, which were associated with a 76.1-, 27.8, 18.4, 17.7-, and 8.3-fold higher risk of adverse outcome, respectively (Table 7). Likewise, serum calcitonin > 8.8 pg/mL was associated with a 20-fold higher probability of a favorable outcome (Table 7).

After stepwise logistic regression analysis, serum osteocalcin > 26.8 ng/mL and PTH > 49.1 pg/mL were the only predictors that remained significant, with area under the ROC (Receiver operating characteristic) curve of 0.73 (95% confidence interval 0.59–0.87) (Table 8 and Table 9).

Positive correlations were found between serum ionised calcium and multivessel CAD (*r* = 0.58, *p* = 0.01) and between serum phosphate and extent of CAC by means of Agatston score (*r* = 0.28, *p* = 0.02). In keeping with these findings, patients with severe CA (SYNTAX score > 21) had higher serum ionised calcium (0.86 (0.38–0.92 mmol/L) versus 0.39 (0.36–0.87) mmol/L, respectively, *p* = 0.018) and alkaline phosphatase (138.0 (60.0–209.0) versus 70.0 (50.0–186.0) mmol/L, respectively, *p* = 0.024) than those with lower SYNTAX score. Likewise, patients with severe CAC (Agatston score > 400) were characterised by a higher serum alkaline phosphatase (0.97 (0.84–1.08) versus 0.88 (0.80–1.03) mmol/L, respectively, *p* = 0.01) as compared with the individuals with mild calcification.

Therefore, preoperative serum concentrations of osteocalcin and PTH were reliably associated with an adverse cardiovascular outcome after 3 years of follow-up. Further, higher serum alkaline phosphatase positively correlated with severity of CA and CAC at the time of hospital admission. Serum ionised calcium and phosphate were also directly associated with severe CA and CAC, respectively, at the same time point.

## 4. Discussion

In the present study, we show that the parameters of mineral homeostasis, in particular osteocalcin, PTH, and alkaline phosphatase, can be useful in prognostication after CABG surgery. Previously, serum PTH > 65 pg/mL was found associated with cardiovascular events in patients with chronic kidney disease (CKD) stages 3 and 4 [18] and with progression of CKD as well as mortality in those with CKD stages 2–4 [19]. Moreover, serum PTH > 6.8 pmol/L was an independent risk factor of CAD in a population-based cross-sectional Tromso study [20].

PTH evinces its effects through PTH 1 receptor which is expressed in cardiomyocytes, vascular smooth muscle cells, and endothelial cells and which level is increased during myocardial ischemia, cardiac fibrosis, and aging [21]. Elevation of serum PTH causes left ventricular hypertrophy [22], myocyte/capillary mismatch [23], and cardiac fibrosis [24]. Excessive PTH disrupts energetic metabolism in cardiomyocytes, leading to a calcium overload [25] and induces carbohydrate metabolism disorders [26] and vascular inflammation [27], correlating with a multivessel CAD, low left ventricular ejection fraction [28], and CAC in patients without CKD [29].

Osteocalcin is a pleiotropic protein regulating glucose and lipid metabolism, nitric oxide release, and endothelial homeostasis depending on its carboxylation status [30]. Opposite to PTH, an association of serum osteocalcin with cardiovascular death is unclear, being U-shaped as well as gender- and age-dependent [31,32,33]. Data regarding the prognostic significance of alkaline phosphatase are also contradictory, varying between revascularisation modalities [34,35,36]. Albeit serum alkaline phosphatase may be also increased in patients with stroke, chronic heart failure, arterial hypertension, type 2 diabetes mellitus, and obesity, a number of studies showed its usefulness as a prognostic indicator during the long-term follow-up of patients with coronary artery disease [35,36] including those with ST-segment elevation MI [37]. In addition, SAP of CCS grade IV, New York Heart Association class IV chronic heart failure, and obesity were among the criteria of exclusion in our study.

Notably, we found bone turnover markers such as PTH, osteocalcin, and alkaline phosphatase associated with an adverse cardiovascular outcome after CABG surgery at a 3-year time point regardless of osteopoenia/osteoporosis, coronary or peripheral atherosclerosis, and CAC. Despite the fact that all indicated proteins are to a certain extent involved in bone resorption, atherosclerosis, and extraskeletal calcification, their predictive value possibly does not depend on respective clinical conditions, rather it is reflective of their combination at a subclinical level. Possible reasons for the discrepancies between the studies include sample size, duration of follow-up, and treatment regimens. We suggest that the preoperative screening of bone turnover markers can be useful in prognostication after CABG surgery due to SAP; however, additional studies on larger samples are necessary for the validation of this approach.

## Figures and Tables

**Table 1:** Clinicopathological features of the patients (*n* = 91).

Feature	Value
Median age (interquartile range)	61 (55–65)
Past medical history of myocardial infarction, *n* (%)	70 (76.9)
Past medical history of stroke, *n* (%)	6 (6.6)
Arterial hypertension, *n* (%)	83 (91.2)
Median left ventricular ejection fraction, % (interquartile range)	57 (48–63)
Type 2 diabetes mellitus, *n* (%)	16 (17.6)
Median body mass index, kg/m^2^ (interquartile range)	24.8 (27.5–29.4)
Smoking, *n* (%)	52 (57.1)
Median glomerular filtration rate, mL/min/1.73 m^2^ (interquartile range)	103 (85–123)
Brachiocephalic artery stenosis > 50%, *n* (%)	15 (16.5)
Median SYNTAX score (interquartile range)	21.9 (20.0–23.8)
Median Agatston score (interquartile range)	781 (624–936)
Osteopoenia, *n* (%)	49 (53.8)
Osteoporosis, *n* (%)	26 (28.6)

**Table 2:** Biochemical parameters regulating mineral homeostasis measured before the CABG surgery (*n* = 91).

Parameter	Median (95% Confidence Interval)	Reference Values (Min–Max)
Total calcium, mmol/L	2.30 (2.25; 2.34)	2.15–2.6
Ionised calcium, mmol/L	0.94 (0.92; 0.96)	1.16–1.32
Phosphate, mmol/L	0.95 (0.92; 0.99)	0.87–1.9
Calcitonin, pg/mL	11.16 (9.03; 13.29)	<30
Parathyroid hormone, pg/mL	31.97 (24.18; 51.81)	16–46
Osteoprotegerin, pg/mL	134.47 (114.61; 154.32)	36.97–101.0
Osteocalcin, ng/mL	21.96 (19.29; 24.63)	9.6–40.8
Osteopontin, ng/mL	7.06 (6.41; 7.71)	0.26–30.0
Alkaline phosphatase, U/L	126.63 (109.44; 143.82)	42–306
Bone alkaline phosphatase, U/L	22.92 (20.60; 25.23)	15–41.3

**Table 3:** Color duplex screening, coronary angiography, and multislice spiral computed tomography results in relation to the adverse cardiovascular outcome after coronary artery bypass graft (CABG) surgery (*n* = 91).

Parameter	Patient Groups		p
	Favorable Outcome (*n* = 70)	Adverse Outcome (*n* = 21)	
Brachiocephalic artery stenosis > 50%, *n* (%)	12 (17.1)	3 (14.3)	0.75
Multivessel coronary artery disease (3 arteries affected), (*n*, %)	48 (68.6)	11 (52.4)	0.17
SYNTAX score > 32, (*n*, %)	17 (24.3)	7 (33.3)	0.41
Syntax score, median (interquartile range)	24 (16-32)	28 (13-34)	0.57
Agatston score > 400, (*n*, %)	42 (60.0)	14 (66.7)	0.58
Agatston score, median (interquartile range)	573 (247–1088)	630 (206–984)	0.78
Osteoporosis, *n* (%)	19 (27.1)	7 (33.3)	0.80
Osteopoenia, *n* (%)	39 (55.7)	10 (47.6)	

**Table 4:** Parameters of mineral homeostasis in relation to the adverse cardiovascular outcome upon CABG surgery.

Parameter	Favorable Outcome (*n* = 70) Median (Interquartile Range)	Adverse Outcome (*n* = 21) Median (Interquartile Range)	*p*
Total calcium, mmol/L	2.28 (2.10–2.4)	2.39 (2.20–2.58)	0.72
Ionised calcium, mmol/L	0.41 (0.37–0.93)	0.41 (0.38–0.90)	0.74
Phosphate, mmol/L	0.97 (0.81–1.05)	0.88 (0.82–1.03)	0.78
Calcitonin, pg/mL	8.51 (6.98–12.57)	7.80 (6.79–8.83)	0.14
Parathyroid hormone, pg/mL	32.62 (25.09–49.07)	45.64 (21.81–62.18)	0.42
Osteoprotegerin, pg/mL	113.58 (61.54–183.92)	70.40 (51.74–109.13)	0.02
Osteocalcin, ng/mL	18.77 (12.67–22.47)	22.75 (16.40–37.66)	0.01
Osteopontin, ng/mL	5.17 (4.42–8.23)	7.87 (4.96–9.47)	0.04
Alkaline phosphatase, U/L	73.5 (49–183)	165 (81–218)	0.03
Bone alkaline phosphatase, U/L	19.62 (14.21–25.71)	21.07 (18.38–25.91)	0.27

**Table 5:** Parameters of mineral homeostasis in relation to the patients with overweight (body mass index ≥ 25 kg/m^2^).

Parameter	Without Overweight (*n* = 69) Median (Interquartile Range)	With Overweight (*n* = 22) Median (Interquartile Range)	*p*
Total calcium, mmol/L	2.29 (2.10–2.40)	2.34 (2.17–2.49)	0.44
Ionised calcium, mmol/L	0.41 (0.37–0.92)	0.66 (0.38–0.91)	0.88
Phosphate, mmol/L	0.94 (0.84–1.06)	0.93 (0.80–1.03)	0.49
Calcitonin, pg/mL	8.28 (6.94–11.26)	9.0 (7.03–13.81)	0.29
Parathyroid hormone, pg/mL	32.62 (24.79–52.33)	38.36 (23.59–54.83)	0.88
Osteoprotegerin, pg/mL	103.73 (60.52–181.66)	88.88 (58.28–128.58)	0.39
Osteocalcin, ng/mL	18.29 (12.83–26.78)	24.67 (18.70–35.07)	0.01
Osteopontin, ng/mL	6.70 (4.48–8.50)	5.02 (4.24–8.91)	0.63
Alkaline phosphatase, U/L	117.0 (56.0–212.0)	88.0 (61.0–179.0)	0.69
Bone alkaline phosphatase, U/L	19.87 (13.65–26.49)	20.88 (17.45–25.41)	0.37

**Table 6:** Parameters of mineral homeostasis in relation to the patients with type 2 diabetes mellitus.

Parameter	Without Type 2 Diabetes Mellitus (*n* = 75) Median (Interquartile Range)	With type 2 Diabetes Mellitus (*n* = 16) Median (Interquartile Range)	*p*
Total calcium, mmol/L	2.27 (2.10–2.48)	2.31 (2.09–2.44)	0.93
Ionised calcium, mmol/L	0.41 (0.37–0.91)	0.63 (0.38–0.95)	0.63
Phosphate, mmol/L	0.93 (0.81–1.05)	1.0 (0.83–1.04)	0.64
Calcitonin, pg/mL	8.38 (6.99–11.87)	8.30 (6.84–11.76)	0.93
Parathyroid hormone, pg/mL	36.27 (24.49–55.91)	30.38 (21.81–33.91)	0.08
Osteoprotegerin, pg/mL	101.54 (59.50–180.29)	80.40 (52.62–171.15)	0.62
Osteocalcin, ng/mL	20.76 (15.55–31.69)	15.01 (12.60–21.30)	0.09
Osteopontin, ng/mL	6.72 (4.54–8.83)	4.87 (4.07–7.33)	0.16
Alkaline phosphatase, U/L	100.0 (60.0–208.0)	130.0 (40.5–175.0)	0.44
Bone alkaline phosphatase, U/L	20.83 (14.18–26.73)	18.43 (15.74–23.33)	0.92

**Table 7:** Variables included into enter logistic regression equations to predict an adverse cardiovascular outcome.

Parameter	Standardised Regression Coefficient (B)	Standard Error	Wald	*p*	Odds Ratio	95% Confidence Interval for the Odds Ratio	
						Lower Bound	Upper Bound
Age > 60 years	−1.926	1.215	2.511	0.113	0.146	0.013	1.578
Past medical history of myocardial infarction	−2.912	1.447	4.053	0.044	0.054	0.003	0.926
Past medical history of stroke	2.579	1.849	1.945	0.163	13.181	0.351	494.306
New York Heart Association class III–IV chronic heart failure	−0.558	1.288	0.188	0.665	0.572	0.046	7.140
Left ventricular ejection fraction ≥ 50%	−2.160	1.441	2.247	0.134	0.115	0.007	1.943
Arterial hypertension	−1.657	1.843	0.808	0.369	0.191	0.005	7.068
Type 2 diabetes mellitus	−1.826	1.646	1.231	0.267	0.161	0.006	4.054
Obesity (body mass index > 30 kg/m^2^)	−0.979	1.012	0.936	0.333	0.376	0.052	2.730
Smoking	2.914	1.451	4.033	0.045	18.437	1.073	316.902
Brachiocephalic artery stenosis > 50%	3.435	1.758	3.817	0.051	31.019	0.989	972.855
SYNTAX score > 32	1.924	1.267	2.307	0.129	6.849	0.572	82.029
Agatston score > 1088	1.045	1.093	0.913	0.339	2.843	0.333	24.231
Osteopoenia/osteoporosis	−1.978	1.327	2.223	0.136	0.138	0.010	1.864
Total calcium > 2.4 mmol/L	2.461	1.276	3.719	0.054	11.719	0.961	142.986
Ionised calcium > 0.9 mmol/L	−1.901	1.556	1.494	0.222	0.149	0.007	3.151
Phosphate > 1.05 mmol/L	−1.708	1.305	1.714	0.190	0.181	0.014	2.338
Calcitonin > 8.8 pg/mL	−3.013	1.343	5.029	0.025	0.049	0.004	0.684
Parathyroid hormone > 49.1 pg/mL	3.326	1.366	5.927	0.015	27.813	1.912	404.592
Osteoprotegerin > 61.5 pg/mL	−2.121	1.372	2.392	0.122	0.120	0.008	1.763
Osteocalcin > 26.8 ng/mL	2.123	1.052	4.072	0.044	8.353	1.063	65.638
Osteopontin > 8.3 ng/mL	4.332	1.879	5.313	0.021	76.082	1.912	3026.805
Alkaline phosphatase > 186 U/L	2.979	1.437	4.298	0.038	17.669	1.177	328.778
Bone alkaline phosphatase > 25.9 U/L	3.431	1.557	4.855	0.068	0.032	0.002	0.685
Constant	0.911	6.743	0.018	0.892	2.488		

**Table 8:** Significant variables predicting an adverse cardiovascular outcome in stepwise logistic regression analysis.

Parameter		Standardised Regression Coefficient (B)	Standard Error	Wald	*P*	Odds Ratio	95% Confidence Interval for the Odds Ratio	
							Lower Bound	Upper Bound
Step 1	Parathyroid hormone > 49.1 pg/mL	1.312	0.521	6.327	0.012	3.712	1.336	10.317
	Constant	−2.998	0.792	14.325	0.000	0.050		
Step 2	Osteocalcin > 26.8 ng/mL	1.142	0.549	4.331	0.037	3.132	1.069	9.181
	Parathyroid hormone > 49.1 pg/mL	1.393	0.543	6.577	0.010	4.028	1.389	11.682
	Constant	−4.657	1.204	14.962	0.000	0.009		

**Table 9:** Prevalence of an adverse cardiovascular outcome and odds ratios in relation to the combinations of risk factors (*р* = 0.00007).

Risk Groups	*n*	Prevalence of Adverse Cardiovascular Outcome	Odds Ratio (95% Confidence Interval)
No increase in parathyroid hormone and osteocalcin	45	13.33%	0.32 (0.11–0.92)
Increase in either parathyroid hormone or osteocalcin	38	23.68%	1.06 (0.40–2.84)
Increase in both parathyroid hormone > 49.1 pg/mL and osteocalcin > 26.5 ng/mL	8	75.0%	13.60 (2.50–74.09)

## Abbreviations

CAC	Coronary artery calcification
CA	Coronary atherosclerosis
PTH	Parathyroid hormone
CABG	Coronary artery bypass graft
SYNTAX	Synergy between Percutaneous Coronary Intervention with Taxus and Cardiac Surgery
CAD	Coronary artery disease
CKD	Chronic kidney disease
SAP	Stable angina pectoris
CCS	Canadian Cardiovascular Society
MI	Myocardial infarction
ROC	Receiver operating characteristic
